# Do We Contribute to Women's Empowerment? Insights From a Nutrition-Sensitive Agriculture Project Implemented in Nong, Laos

**DOI:** 10.1177/03795721241293547

**Published:** 2024-11-06

**Authors:** Manon Gerber, Daniëlle M. Bon, Bounthanom Bouahom, Jacqueline E.W. Broerse, Dirk Essink

**Affiliations:** 1 Faculty of Science, 1190 Vrije Universiteit Amsterdam and Amsterdam Public Health Institute, Amsterdam, The Netherlands; 2 Rural Economy, 343025 National Agriculture and Forestry Research Institute, Vientiane, Laos

**Keywords:** women's empowerment, food and nutrition security, nutrition-sensitive agriculture, Lao PDR

## Abstract

**Background:**

The gender-gap in power is still persistent around the globe. Nutrition-Sensitive Agriculture (NSA) interventions have been implemented to increase women's empowerment as a goal in itself, and as a pathway to food and nutrition security (FNS). However, contradicting evidence exists on whether the interventions, besides food security, realize women's empowerment. Furthermore, the concept of women's empowerment has different meanings across different cultures, regions, and countries.

**Objective:**

To assess the understanding of, and perspectives on, women's empowerment among different stakeholders in the context of an NSA project in Laos, and to assess whether this project contributes to women's empowerment and FNS.

**Methods:**

Semistructured interviews were conducted with local implementers of NSA interventions (n = 13) and senior program managers and advisors (n = 5). Six focus group discussions were conducted with community members (n = 46).

**Results:**

Our findings reveal that community members had a materialistic understanding and local implementers an instrumental understanding, in contrast to senior program managers and advisors, who had an egalitarian understanding of women's empowerment. The level of women's empowerment in Nong was considered low by all respondents. Furthermore, respondents reported that the NSA interventions had a positive impact on FNS, but not on women's empowerment.

**Conclusions:**

In a community in which both women's empowerment and FNS are low, working with women may contribute to FNS, but not necessarily contribute to women's empowerment. Nevertheless, from an emic perspective, women do feel more empowered as the interventions contributed to increased household capacity to address FNS.

**Plain language title:**

Does our project lead to having enough food and improved women’s status in communities living in rural, mountainous areas in Laos?

## Plain language summary

Many women around the globe, particularly in low- and middle-income countries, belong to the most disempowered groups of society as they experience economic and social disadvantages relative to men. Previous studies have demonstrated that empowering women can result in increased food and nutrition security (FNS), as well as economic growth. Therefore, this study investigated whether Nutrition-Sensitive Agriculture (NSA) projects improved women's empowerment (WE) and FNS in remote villages in Laos. The study's findings indicated that while NSA projects did increase FNS, they had no positive impact on WE which was considered to be low by all participants and even increased the already high workload of women. The study also discovered that community members, grassroots implementers of the NSA interventions, and senior program managers and advisers (SPMAs) all had different conceptual understandings of WE, highlighting the conceptual unclarity of WE. SPMAs perceived WE as working toward (gender)equality and moving away from gender-based activities. This was in contrast to community members and local implementers, who understood WE as the capacity to perform well in their gender-based tasks. Consequently, we think we are all working toward the same construct of WE, but due to the conceptual unclarity we are all working toward other goals which result in the risk of “using” women rather than working toward the end goal of WE. Therefore, our study suggests that conceptual clarity of the concept you are working on in a particular project context, in this case WE, is essential for implementing interventions based on it.

## Highlights

Conceptual unclarity about the concept of women's empowerment (WE) exists which results in working toward different goals with the risk of “using” women rather than working toward WE. Consequently, women's contribution to the Nutrition-Sensitive Agriculture (NSA) project did improve food and nutrition security (FNS), nevertheless, it did not improve WE, but rather adds to their already high workload.

## Introduction

Many women around the globe are among the most disempowered groups in society, due to impeding socioeconomic factors, and inequality of power compared to their male counterparts. This is particularly evident in low- and middle-income countries (LMICs).^1,2^ Therefore, WE is at the core of Sustainable Development Goal (SDG) 5, gender equality.^3^ Moreover, WE is not only a goal but is also a means to reach other SDGs. Women in LMICs form the backbone of their society and empowering them contributes to realizing goals such as economic development and food and nutrition security (FNS).^4–6^

Previous research has recognized this so-called synergy, where improving both FNS and gender equality together could lead to greater results than focusing on both goals separately.^7,8^ Women contribute greatly to FNS by engaging in multiple agricultural activities, ranging from harvesting and transplanting, to taking care of cattle.^5^ WE can lead to better childcare and feeding practices^9^ and is even positively associated with early cognitive development and child growth.^10^ Previous research also showed that empowering women results in increased agricultural production, and improved FNS.^11–13^

Nutrition-Sensitive Agriculture (NSA) is one approach that aims to improve FNS, specifically the nutritional status of children under 5. It works through a combination of pathways including increased WE, but also increased and diversified production and higher income from agricultural produce.^14^ A standardized Women's Empowerment in Agriculture Index (WEAI) has been developed to measure WE and inclusion in the agricultural sector. It measures WE across 5 domains in agriculture and the relative empowerment gap with respect to their male counterparts.^15^

However, the influence of NSA interventions on WE is not univocal. Multiple studies have found that NSA interventions could add more work to the already high burden that women experience in agriculture.^16,17^ This high workload could eventually lead to lower FNS, because the time and energy women need for the interventions is at the expense of other tasks like taking care of their children, preparing food, or other health behaviors.^18–20^ Furthermore, a previous review identified both positive effects of NSA interventions on WE and negative effects, due to the context specificity of cultures, including different levels of empowerment and different barriers, challenges, and needs.^21,22^

The contradictory evidence could partly be explained by the lack of clarity related to the concepts, how it affects NSA projects and how it is operationalized. The concept of WE is not univocally understood and may have different meanings across different cultures, regions, and countries.^23^ Therefore, to implement interventions based around WE, an understanding of the meaning of it within a particular (project) context is needed.

This study aims to assess the understanding of, and perspectives on, WE among different stakeholders in the context of an NSA project in Laos, and assesses whether this project contributes to WE and FNS.

We conduct a case study by examining the emic and etic perspectives on WE in the rural mountainous area of Nong, Savannakhet province in Laos. The empowerment of Lao women is considered low, particularly in rural communities gender inequality persists.^1^ The communities living in Nong rely on their agricultural production, in which women play a crucial role as producers of food, managers of natural resources, income earners, and caretakers of the household.^24^ NSA interventions have been implemented in the villages in Nong between December 2016 and June 2021 both to improve WE as a means to FNS but also as an end in itself.

## Methods

### Study Design

This study followed a qualitative phenomenological approach, using an emergent design.^25^ This approach was chosen because it allows for an in-depth exploration of stakeholders’ personal experiences and perspectives on WE, which is essential for understanding how the NSA project in Laos contributes to WE and FNS. This study consisted of both semistructured interviews and focus group discussions (FGDs) to elucidate and discuss the lived experiences, to increase the quality of the results by data triangulation and to obtain a more comprehensive view of WE.^26^

### Study Setting

This study was conducted between April and July 2020 and is part of the project called “Scaling up NSA in mountainous areas in Laos and Vietnam.” The project was a collaboration between the local government, 2 NGOs (MCNV & CODA) in Laos, the Lao Tropical and Public Health Institute, the National University of Laos, and the Vrije Universiteit Amsterdam. NSA interventions were implemented from December 2016 to June 2021 in 20 remote villages in the district of Nong, Savannakhet. The interventions aimed to contribute to NSA through pathways of food production, nutrition-related knowledge, agricultural income, strengthening of local institutions, and WE. The NSA interventions included: food production mainly for own consumption (vegetable home gardens, catfish and frog ponds, and the holding of lay-egg chickens), training programs on Water, Sanitation, and Hygiene (WASH) and other topics such as childcare, health, nutrition, and agriculture and cooking demonstrations.^27^ The WASH training focused on promoting hygiene practices and ensuring access to clean water. The local implementers focused mainly on involving female family members for the implementation.

Nong is a district where poorly resourced, ethnic minorities live, resulting in a low FNS and high stunting (72.8%) and wasting rates (10.4%) among children under 5.^28,29^ For this research, 4 villages (Phatouy Ngai, Kenglin, Along and Por) were selected based on their diverse geographical locations and to represent households from different socioeconomic status.

### Study Population and Sampling Procedure

Three different study populations were included in this study: senior program managers and advisors (SPMAs); local NSA implementers; and community members, to understand both the emic and etic perspectives on WE. A total of 64 participants were consulted in this qualitative study.

Individual interviews were conducted with 5 SPMAs, all women, and 13 local NSA implementers (4 women and 9 men). Purposive sampling was used to target both field implementers and coordinators of different programs and offices in Nong related to NSA. This included the agricultural programs of the NSA, the health office, nutrition office, and women's union in Nong. The SPMAs that were targeted held a senior role in MCNV or other organizations or ministries related to agriculture and gender development. Thereafter, a variety of local implementers working on one of the NSA interventions in Nong, SPMA working for MCNV and other organizations related to gender equality or WE in agriculture in Laos, were selected until data saturation was reached.

Members from the community level were purposely sampled for FGDs. In total, 6 FGDs with 6 to 9 participants were organized. Of these FGDs, 4 FGDs were conducted with women (n = 31) and 2 with men (n = 15). Women were included if they participated in at least one of the NSA interventions implemented in the area, were between the age of 15 and 60, and were either head of the household or married to the head of the household. Men were included if they were from the same household as the women and participated in at least one of the NSA interventions. To make sure that both men and women were encouraged and dared to speak up, separate FGDs were conducted for men and women. This was done as previous research showed that women speak much less in group settings when men are present.^30^

### Data Collection Instruments and Process

The interview and FGD guide were developed using the domains of WEAI.^15^ The WEAI was used to improve the understanding of WE and the perspectives on women's level of empowerment. The WEAI consists of 5 domains to define the level of WE in agriculture, including:^15^

- *Production*: the ability of making decisions regarding agricultural activities.
- *Resources*: the access to, and decision-making over, productive resources.
- *Control over income*: the ability to generate income and the ability to spend it freely.
- *Leadership*: group membership and public speaking.
- *Time allocation*: the workload women carry and their time availability for other activities in leisure time.

To increase the validity, pilot interviews and FGDs were conducted.^31^ Semistructured interviews were conducted to understand participant's perspectives on the level of empowerment in Nong, their understanding of empowerment and the impact of the NSA interventions on WE. Interviews were conducted using Zoom due to COVID-19 restrictions at that time. Depending on the preferences and abilities of the participants, interviews were conducted in Lao or English. All interviews were transcribed in the language in which they were conducted, and the Lao transcripts were translated to English.

After the interviews, FGDs were conducted to explore and gain understanding of opinions and issues from the perspectives of and interactions between the community members.^32^ For this, an FGD guide was developed based on the results of the interviews and the domains of the WEAI. The WEAI domains of Resources and Production were combined into a new domain, decision making, as participants not only frequently referred to decision-making power when discussing these areas, but also mentioned other facets of decision making, like joint-decision making, and decision making in the household. In addition to the WEAI themes, other topics covered included the understanding of WE, groups and support networks and realizing unfairness. No local definition of WE existed, therefore, we explored the local definition by asking both men and women to describe and tell a characteristic of a “strong woman” and a woman as a “role model” within or outside the community.

FGDs were conducted in local language and translated to Lao and thereafter translated to and transcribed in English. FGDs with community members were conducted face to face within Nong.

### Data Analysis

Data was analyzed using the analytic software program ATLAS.ti (version 8.4.4).^33^ Interviews were analyzed iteratively, by coding them immediately after transcription and performing a rough analysis of the data after 2 interviews were coded. FGDs were analyzed thematically afterwards. All data were analyzed to identify recurrent themes, similarities, and differences across interviews and FGDs. The process of analyzing was discussed weekly with 2 other researchers to increase confirmability. A codebook was created based on existing literature such as the concepts of WEAI and an additional inductive approach to include context-specific factors. As described in the data collection section, the WEAI domains of Resources and Production were merged into a new domain, decision making.

### Ethical Considerations

This study obtained ethical approval by the National Ethical Committee of Health Research of Laos, No. 099/NECHR. Verbal informed consent was given by all participants. Participation was voluntary and participants were allowed to refuse to answer any questions and to leave the study at any time without explanation.

## Results

### Understanding of Women's Empowerment

We have distinguished 3 different understandings of WE: the materialistic understanding as expressed by community members; the instrumental approach as expressed by local implementers, and a more egalitarian understanding as expressed by SPMAs.

#### Understanding of Community Members

During the FDGs, women reported that they felt empowered when they were able to perform their domestic and agricultural tasks well, were able to generate an income and good production rate and had the resources to do so. They emphasized that they needed to have materialistic means such as a large home garden or multiple farming activities in order to perform their tasks well and to be food secure. They hardly referred to any decision-making power. The materialistic approach of WE by the community is explained by the following quote:

*“An empowered woman is a woman who has a vegetable home garden, upland farming, paddy field.”* (Women-FGD1)

This was similar to the perspective of male community members during the men FDGs, who mentioned that WE means that women can perform their tasks well.

*“Women's empowerment is women who do housework and take lead to do [household] work.”* (Men-FGD1)

On the question “when is a woman empowered?”, most women in the FGDs said and confirmed that they see WE almost synonymous with being able to do gender-based tasks. For example, one woman indicated to the question:

*“Women [who are empowered] have multiple tasks such as doing upland farming, paddy field, weaving, taking care of children, feeding chicken and pig and many other tasks.”* (Women-FGD4)

#### Understanding of Local Implementers

In line with the community level respondents, almost all local implementers agreed that being empowered revolves around women being able to perform well in their culturally determined gender-based tasks. Though, they had a more instrumental approach as they put more focus on the capability and knowledge that women needed to perform these tasks rather than having the means to do so or the production it generated. According to the perspective of most local implementers, women were empowered when they could choose how and when to do their tasks and had the knowledge to perform them well. This is illustrated by the following quote of a local implementer:

*“To make a woman capable. Like I told you, [we should] make a woman capable of improving their household income, to improve the food security in the household and also to enable women to manage their household.”* (R2-local implementer)

#### Understanding of SPMAs

None of the community members and local implementers raised issues as “equal opportunity” or “equality” between men and women. In contrast, SPMAs held a more egalitarian view of WE. All SPMAs viewed WE as being aware of your needs, the ability to act upon them and foremost, having equal opportunities in life as men. Having equal opportunity meant having the same access to information or being able to obtain the same level of education as men, rather than focusing on physical means or knowledge and capability within gender-based tasks. This is illustrated by following quotes of different SPMAs:

*“Women's empowerment means they should have equal opportunity to access information, that they have a sufficient educational level, that they are aware of their needs and that they can negotiate and have a voice.”* (R9-SPMA)

### Domains of WE

The perspectives on WE are further detailed in relation to FNS using 4 domains of WE that were identified during the data collection and are based on the domains of WEAI: time allocation, decision making, control over income, and leadership.

#### Time Allocation

Participants of the focus groups mentioned time allocation foremost regarding work and daily tasks and often did not discuss leisure time. Respondents on all levels agreed that the workload of women is very high. During the FDGs, women reported that they spend their day working in upland farming, collecting firewood and bamboo shoots on the way back to the village, while taking care of their children and household chores simultaneously. Before men start their day, women have already been up for several hours in order to be able to complete their daily tasks in time. This high workload of women is illustrated by the following quotes of a local implementer and community member:

*“I’m surprised when I see the women in the village wake up at 5 am or sometimes 3 or 4 am to do the hand rice mill. After waking up they do hand rice mill, clean the house, take care of the children, and go watering the vegetable home garden. Then, they will cook for the husband after the husband gets up.”* (R7-local implementer)

*“It is not good but we have to do [the tasks] because we would like to have food to eat. Actually, we have many tasks to do and we are tired.”* (Women-FGD4)

Traditional gender roles were ubiquitous in Nong resulting in women having almost 3 times as many tasks as men (see also Table 1). The high workload of women compared to men is also illustrated by the following quote:

*“Women's workload is exponentially higher than men, they are doing everything: they are doing child raising, they’re doing the cooking, the cleaning, the gardening (…) they are doing the book of the workload!”* (R4-SPMA)

**Table 1.** Gender Tasks in Nong and Actual Workload Reported by Community.

Gender tasks	Men tasks	Women tasks	Actual workload women
Cooking		X	X
Collecting food		X	X
Collecting firewood		X	X
Household chores		X	X
Hand rice mill		X	X
Hunting and fishing*	X		X
Slash and burn activities	X		X
Taking care of children		X	X
Taking care of home garden		X	X
Upland farming activities	X	X	X

* *Women do part of the fishing but in a different style than men. Where men can take the boat, women stand in the water for fishing*.

Although some men were reported to support women in a few of their gender-based tasks, such as collecting firewood, all women mentioned that they would like men to work more to reduce their workload as they saw the work division as unfair. Nevertheless, they do not think this will improve anytime soon due to the traditional gender roles.

*“If we do not do it then we do not have any food to eat. If we are waiting for our husband then we will have nothing. We won’t have an upland rice field or home garden if we practice or live like our husband.”* (Women-FGD4)

The work of men mainly consisted of specific tasks that were considered physically hard by most respondents. Some local implementers mentioned they saw only a handful of men taking care of the children from time to time. However, women are also occupied with men-based tasks, such as hunting and fishing, slash and burn activities, and upland farming (Table 1).

Both women and men agreed during the FGDs that the current situation in Nong with regard to workload is unfair. Nevertheless, almost all women did not dare to ask their husbands for help. This is illustrated by the following quote:

*“We are not satisfied but what should we do? We could not ask the husband to do more because we are afraid he will leave us and will have another girl.”* (Women-FGD2)

Although male community members reported that the current division of workload was unfair as well, most of them mentioned that they lacked the knowledge or skills to help their wife and argued that it is inappropriate to help. They often did not feel like working, but even if they wanted to help it was not socially accepted in their culture to participate in women's tasks. Some men explained that they had never seen a man doing a woman's task, therefore feeling unsure whether they could perform these tasks:

*“We could not do hand rice mill and water fetching because we are shy and other friends will laugh at us. The friends will think that we are scared of women.”* (Men-FGD1)

#### Decision Making

Nearly all respondents mentioned that in most cases men held the decision-making power with regard to production and resources. It became apparent that, in general, joint decision making was limited, although views differed between respondents. On the community level, both women and men mentioned there is no real joint decision making.

*“If the men decide then women have to follow. In case of chicken raising if the women would like to do something but the men disagree then women could not do it because women could not build the chicken house.”* (Women-FGD2)

Some men mentioned that they did have joint planning with their spouses; still, men had the final say in decisions.

*“We make the planning together” […] then husband makes the decision and the wife follows.”* (Men-FGD2)

However, it did become apparent that there were a few things that some women could decide themselves. Most women and implementers mentioned that women are often the ones to make decisions regarding childcare and feeding practices.

#### Control Over Income

During both FGDs with men and women, it became clear that men are in control over income. Men decide how much money is spent and what it is spent on. Most respondents mentioned that women needed permission from their husband when they wanted to buy something. There were only 2 occasions mentioned by women during the FGDs when they did not need permission. First, when they wanted to buy something small that could be used in the household and home garden, such as soap, salt and seeds, which made them able to decide what crops to grow:

Women: “*The husband takes lead on land preparation for the home garden, but the wife decides which crops to grow.”*

Interviewer: “*So men just prepare the land for you, but he does not know which crop that you will grow right?”*

Women: “*Yes, that's right. Women take lead.”* (Women-FGD2)

Second, when they were able to buy goods from their own income. Some local implementers mentioned that they saw a change happening in control over income now women are able to earn their own money. However, it became clear that women do not earn much, making them still largely dependent on their husband's income. This is illustrated by following quote from the women's FGDs:

*“If we can earn money on our own then we can spend without asking permission from our husbands. But if the husband earns money […] we have to ask permission to spend it.”* (Women-FGD1)

While men are the ones who control the income, men often share a part of their income with women for safekeeping. Several women mentioned this could be either to save up for an emergency or simply to make sure that their husband does not spend his income on unnecessary things. This was confirmed in the FGD with men:

*“The wife controls income. If the husband controls income, then the husband will spend it on alcohol.”* (Men-FGD1)

#### Leadership

All respondents indicated that women cannot take a lead role in their community. In relation to the specific NSA activities, all women mentioned having limited opportunity to participate and share their opinion in meetings at village and community level. Three reasons were mentioned for the limited opportunity to participate. First, women rarely get invited to meetings. In general, the head of the household, which is most often the husband, gets invited by the village head to join the meeting. This made women only able to join when their husband was sick or unavailable. Second, when women were invited to the meetings, they still needed permission from their husband to join. This was also the case for NSA related meetings and training. When asked why women were not always allowed to join the meetings, a man responded:

*“Because they ask men to come to the meeting. Men could talk and discuss during the meeting, but the women are not much talking and a woman has many household tasks: she has to take care of the children and has to cook at home.”* (Men-FGD1)

Third, it became apparent that even if they would have permission to participate in meetings, they often did not have enough time to join, primarily because of their high workload. Nevertheless, local implementers’ view differed from the SPMAs and community on why women participation was low. Most SPMA mentioned that women have no time to join, while most local implementers mentioned that women were too shy to join instead of the lack of opportunity to join:

*“In the community where I have been, they are shy, they do not say anything or give any comment, they mainly listen because of their capacity.”* (R3-local implementer)

With regard to public speaking, the majority of the respondents agreed that women were not likely to speak up during meetings. Furthermore, they were also not likely to speak up to their husband or other authorities. Some women mentioned not feeling capable or knowledgeable enough to speak up. However, most women mentioned that they were scared that their husband, neighbor or even the village head would get angry at them, as one woman said during the FGD:

Interviewer: “*Why are you nervous to speak it out?”*

Women: *“Because we are wondering if the other person will get angry at us.”* (Women-FGD4)

In the case of group membership, most implementers stressed the importance of having women's groups. Local implementers mentioned that women felt more comfortable speaking up when there were more women around. However, almost all women mentioned not participating in any group, because women groups were not established in their village as illustrated by the next quote:

Interviewer: “*How about a women group? Do you have any women groups in your village such as weaving groups, production groups?”*

Women: *“We do not have any groups. We are weaving individually.”* (Women-FGD1)

### Effects of NSA Interventions

Perceptions of the impact of the NSA interventions on FNS were mostly positive for all groups consulted. Local implementers stated that NSA interventions made women more knowledgeable on how to cook and grow crops, which in turn increased their production. In the FGDs with women this was confirmed as many households had more food to eat and to sell to generate an income. Therefore, most women wanted to participate in as many interventions as possible:

*“I want to participate in many interventions, such as frogs and fish. And they laugh at me because I am very old, but I would like to do many interventions. I want to participate in more interventions in order to eat and sell.”* (Women-FGD4)

Although the NSA interventions seemed to increase FNS, the effect on WE is not so clear. As mentioned before, men rather than women often joined the meetings and were subsequently also the ones making decisions regarding the interventions, while women were the ones who executed the interventions. This is illustrated by the following quotes of an SPMA:

*“It is really challenging in some ways trying to push women's empowerment. Women don’t have the time to learn about empowerment, they are so busy! And then a lot of the projects that we want to do involve women, but they don’t have time to do that.”* (R5-SPMA)

Women did, especially when they were able to attend meetings, increase their capacity, but it should be noted that most interventions ultimately were implemented by women, which further increased their workload, without increasing their decision-making power.

*“In general, women mainly do the task. Men are the ones who make the decision to join interventions, but women are the ones who implement.”* (R8-local implementer)

## Discussion

Our findings reveal that there is a major difference in understanding of WE among SPMAs, local implementers and community members, illustrating the conceptual unclarity of WE. Community members generally have a materialistic understanding of WE; grassroot-level implementers have an instrumental understanding; and SPMA have an egalitarian understanding of WE (Table 2). Consequently, community members and local implementers actually reinforce gender roles by aiming to make women capable within their gender-based tasks, instead of striving toward gender equality. From all perspectives WE was perceived as low.

**Table 2.** Key Difference in Understanding of WE.

Stakeholder	Understanding of WE
Community members	Materialistic understanding: women are as empowered when they are able to have the physical means and resources to perform well in their gender-based tasks
Local implementers	Instrumental understanding: women are empowered when they have the knowledge and capability to perform well in their gender-based tasks
SPMAs	Egalitarian understanding: women are seen as empowered when they have equal opportunities in life as men. Having equal opportunity means having the same access to information or being able to obtain the same level of education as men. Thus, moving away from the current gender-based tasks

Abbreviations: SPMA, senior program managers and advisors; WE, women’s empowerment.

From an egalitarian perspective, in our study NSA related improvements hardly had an impact on WE and generally increased their already high workload. The egalitarian perspective resembles conceptualizations in literature in which WE is interlinked to gender equality.^3^ From this perspective, women in our study have low decision-making power regarding resources, almost no control over income or their workload and experience almost no opportunity to speak up or join groups. Moreover, the instrumental approach of the local implementers in our study resulted in an additional burden to the already high workload of women as the underlying gender roles, such as seeing childcare as women's responsibility, were not addressed.^34^ Thereby reinforcing already existing gender roles in which women do most of the farm work, but also tend livestock, and perform off-farm activities such as collecting firewood, cooking, and taking care of children and their household.^35^ However, the NSA interventions did improve women's control over money and therefore their decision-making power with regard to agricultural practices. From a materialistic perspective of the community in Nong, several improvements were seen in WE; the NSA interventions contributed to more household resources, which made women generate more agricultural produce and resulted in improved nutritional status. In line with our study, Kumar et al^36^ also found that NSA interventions did improve women's agriculture empowerment by improving decision-making power for agricultural tasks and increased income in agricultural production but did not have a positive effect on women's decision-making power for nonagricultural topics and perception of gender equality.

Somewhat parallel to the discussion of empowerment, the community mentioned unfairness in the division of gender-based tasks. However, they did not link this unfairness to the concept of WE. Research on critical consciousness suggests that as communities progress through the states of transitive consciousness they better understand and challenge social structures.^37^ During NSA activities, in which community members were stimulated to become aware of the differences in workload between men and women, community members recognized this was unfair. However, this doesn't mean that this recognition of unfairness in this context was translated into challenging social structures.

When not having clarity on the concept of WE, different perceptions and understandings exist when a woman is empowered. For example, previous research found that according to the WEAI, women with permanently out-migrated husbands were rated as empowered, while they were seen as the least empowered when rated by other women and self-rated.^34^ This again illustrates that as there is no agreement on the concept of WE, different meanings are attached toward the concept which results in different views on what interventions are needed, instead of cohesively working toward the same end goal of empowering women.

In our study, the way WE is understood from a materialistic approach is hard to distinguish from other development goals such as FNS and poverty: when a woman has the means to generate food and income, she feels empowered. Although both WE and having enough resources are important, we should be careful that the goal of WE is not equated to the other SDGs and that not the same thing is measured for different goals (such as that having more food production automatically leads to an improvement in WE). Still, these goals might be synergistic.^7,8^

Nevertheless, WE from an egalitarian understanding will be hard to realize if there will not be a shift in the attitude of the local implementers and community members toward gender roles. In contrast to most Lao-Tai women and some ethnic minorities, most ethnic minorities in Nong follow a patriarchal system. In this patriarchal system there are prescribed gender roles where women are inferior to men while a matri-social kinship pattern promotes gender equality and a higher status for women.^38^ This study confirms the “unfair” gender roles in the context of Nong. This gives them limited opportunities for development towards gender equality because their gender tasks are deeply embedded in their society. This suggests that a cultural shift needs to take place before gender roles can change in Nong.

However, to reach critical consciousness on gender equality it is likely that this is facilitated when some materialistic needs are satisfied. This is in line with the self-determination theory as women feel more capable and competent when they have a basic level of resources.^39^ Still, gender equality can be realized when materialistic needs are not fully satisfied, since these processes are often not hierarchal and overlap. Therefore, it is advised to work on both aspects: the critical consciousness on gender equality but also improving the materialistic position of women and households.

### Strengths and Limitations of the Study

By using a qualitative research design, an in-depth understanding of not only the level of WE based on the WEAI in the context of Nong could be generated, but also their perspectives on the meaning of and their perceived level of WE. While previous studies have mostly focused on improvement of WE, this study was able to examine WE on a deeper level by understanding the different definitions of WE from both the emic and etic perspective. In addition, not only the perspectives of women in the community, but also the perspectives of men were included in this study which resulted in a more accurate image of the current situation and perspectives on WE in Nong. Moreover, coherence in perspectives on WE was found within the different stakeholder groups (SPMAs, local implementers, and community members). Next, the use of multiple data-collection methods strengthens the credibility of this study. Moreover, multiple researchers played a role in gathering, transcribing and analyzing the data to increase validity.

A limitation of this research was that the community members were from ethnic minorities that spoke different languages, which might have resulted in inaccuracies in translation. Moreover, no word for WE existed in the local language. This made translation of the term hard and therefore could either have been misunderstood or been too suggestive when explaining the term. To reduce the risk of translation and interpretation bias, the term WE was discussed with a local translator and all English transcripts were read by a bilingual researcher, to cross check the translation. Moreover, in this study an egalitarian perspective of WE was taken for the definition, while conceptual unclarity about the term WE exists. This may have affected not only the interpretation of local implementers, but also by extension community members/partners during the interventions period. Furthermore, some data was collected online because of the COVID-19 disease measures during the first outbreak. Lastly, the findings of this study are highly context specific. Although the context specifics may not be directly relevant to all settings, we do hypothesize that the issue of conceptual unclarity in relation to WE is critical throughout marginalized communities and communities with gender inequalities.

### Recommendations

Using the insights of this study, a couple of recommendations regarding future research and for improving the policies to achieve WE and FNS in Nong, taking into account the cultural context, were identified. First of all, our study highlights that conceptual clarity on WE within projects is critical. Project teams may assume they are all working towards the same construct of WE, but due to the conceptual unclarity they may be working toward other goals which result in the risk of “using” women rather than working toward the end goal of WE. Furthermore, it should be discussed whether the egalitarian perspective of WE is the most appropriate approach to achieve empowerment of women in all settings at least in the short term. For example, there are limited opportunities for personal development, access to information and education in Nong. While striving for equal opportunities and access is an important goal, it seems that for now the community in Nong is more in need of resources, knowledge and capability to reduce poverty than increasing gender equality. Therefore, it is advised to further research the different approaches of WE to gain a deeper understanding of WE as a pathway to FNS. In the long term, it is recommended to focus more on WE as an end in itself rather than a means. This will help to prevent interventions from continuing to reinforce existing gender roles. The awareness of the unfairness of both males and females in Nong will be the breeding ground for a strong movement toward WE as an end in itself. This critical understanding is a precondition to initiate positive change and for future interventions to effectively promote gender equality. Previous research in South Africa showed that techniques such as rooting discussions, probing, and role-plays, can build critical consciousness to promote WE.^40^ To make these techniques effective, it is important to create women's groups. These groups can provide a space for women to discuss issues and share experiences related to their workload, which stimulates greater awareness. This will be helpful as women might remain silent when men are present. In addition, it will also be helpful for implementers to reach and engage with women.^41^

Moreover, previous research found that different domains and indicators of the WEAI are important in different settings to reach FNS due to the context specificity of gender roles.^23^ Therefore, it might be necessary to conduct more research to understand which domains of WE matter most for improved outcomes in FNS before implementing interventions. In this study, it was found that especially the workload was something that women perceived as unfair which shows that this might be one of the most promising areas for (policy) interventions. Nevertheless, more research is needed on how men can be convinced to support women taking into account their traditional gender roles. A possible approach for this is participatory action research (PAR) to ensure that the perspectives, cultures, priorities, and concerns of those that are most affected—the community members—are integrated in the solution. Moreover, for the other dimensions of WE it might be necessary to first improve the realization of unfairness within the community.

## Conclusion

Our findings reveal that community members and grassroot level implementers have a different understanding of WE compared to SPMAs. The community and grassroot level implementers had a materialistic and instrumental understanding of WE with no reference to equality between men and women, while SPMAs did emphasize the importance of gender equality. Overall, the level of WE in Nong was perceived as low by all participants. Although the NSA interventions did not improve WE based on the WEAI, most respondents reported that it did improve FNS. Therefore, our findings challenge the assumption that WE can be used as a means to achieve FNS. Nevertheless, from a materialistic perspective, an increase in WE was seen, as this study indicated that the NSA project contributed to household resources and capacity to address FNS. Therefore, NSA interventions can be considered a stepping stone toward reaching WE.

## Appendix 1: List of Acronyms

FGD: focus group discussion
FNS: food and nutrition security
Lao PDR: Lao People’s Democratic Republic
LMIC: low- and middle-income countries
MCNV: Medical Committee Netherlands–Vietnam
NSA: Nutrition-Sensitive Agriculture
SDG: sustainable development goals
SPMA: senior program managers and advisors
WASH: water, sanitation, and hygiene
WE: women’s empowerment
WEAI: Women’s Empowerment in Agriculture Index
